# Adaptation to the shallow sea floor environment of a species of marine worms, *Oligobrachia mashikoi*, generally inhabiting deep-sea water

**DOI:** 10.1038/s41598-023-33309-6

**Published:** 2023-04-18

**Authors:** Shouzo Ogiso, Kazuki Watanabe, Yusuke Maruyama, Hiroshi Miyake, Kaito Hatano, Jun Hirayama, Atsuhiko Hattori, Yukina Watabe, Toshio Sekiguchi, Yoichiro Kitani, Yukihiro Furusawa, Yoshiaki Tabuchi, Hajime Matsubara, Mana Nakagiri, Kenji Toyota, Yuichi Sasayama, Nobuo Suzuki

**Affiliations:** 1 Noto Marine Laboratory, Institute of Nature and Environmental Technology, Kanazawa University, Ogi, Noto-cho, Ishikawa 927-0553 Japan; 2 Department of Biology, College of Liberal Arts and Sciences, Tokyo Medical and Dental University, Ichikawa, Chiba 272-0827 Japan; 3 Department of Clinical Engineering, Faculty of Health Sciences, Komatsu University, Komatsu, Ishikawa 923-0961 Japan; 4 School of Marine Biosciences, Kitasato University, Sagamihara, Kanagawa 252-0373 Japan; 5 Division of Health Sciences, Graduate School of Sustainable Systems Science, Komatsu University, Komatsu, Ishikawa 923-0961 Japan; 6 Department of Pharmaceutical Engineering, Faculty of Engineering, Toyama Prefectural University, Kurokawa, Toyama 939-0398 Japan; 7 Life Science Research Center, University of Toyama, Sugitani, Toyama 930-0194 Japan; 8 Noto Center for Fisheries Science and Technology, Kanazawa University, Ossaka, Noto-cho, Ishikawa 927-0552 Japan

**Keywords:** Animal physiology, Animal behaviour

## Abstract

Beard worms from the family Siboglinidae, are peculiar animals and are known for their symbiotic relationships with sulfur bacteria. Most Siboglinids inhabit the deep-sea floor, thus making difficult to make any observations in situ. One species, *Oligobrachia mashikoi*, occurs in the shallow depths (24.5 m) of the Sea of Japan. Taking advantage of its shallow-water habitat, the first ecological survey of *O. mashikoi* was performed over a course of 7 years, which revealed that its tentacle-expanding behavior was dependent on the temperature and illuminance of the sea water. Furthermore, there were significantly more *O. mashikoi* with expanding tentacles during the nighttime than during the daytime, and the prevention of light eliminated these differences in the number of expending tentacles. These results confirmed that the tentacle-expanding behavior is controlled by environmental light signals. Consistent with this, we identified a gene encoding a photoreceptor molecule, *neuropsin*, in *O. mashikoi*, and the expression thereof is dependent on the time of day. We assume that the described behavioral response of *O. mashikoi* to light signals represent an adaptation to a shallow-water environment within the predominantly deep-sea taxon.

## Introduction

**Figure 1:**
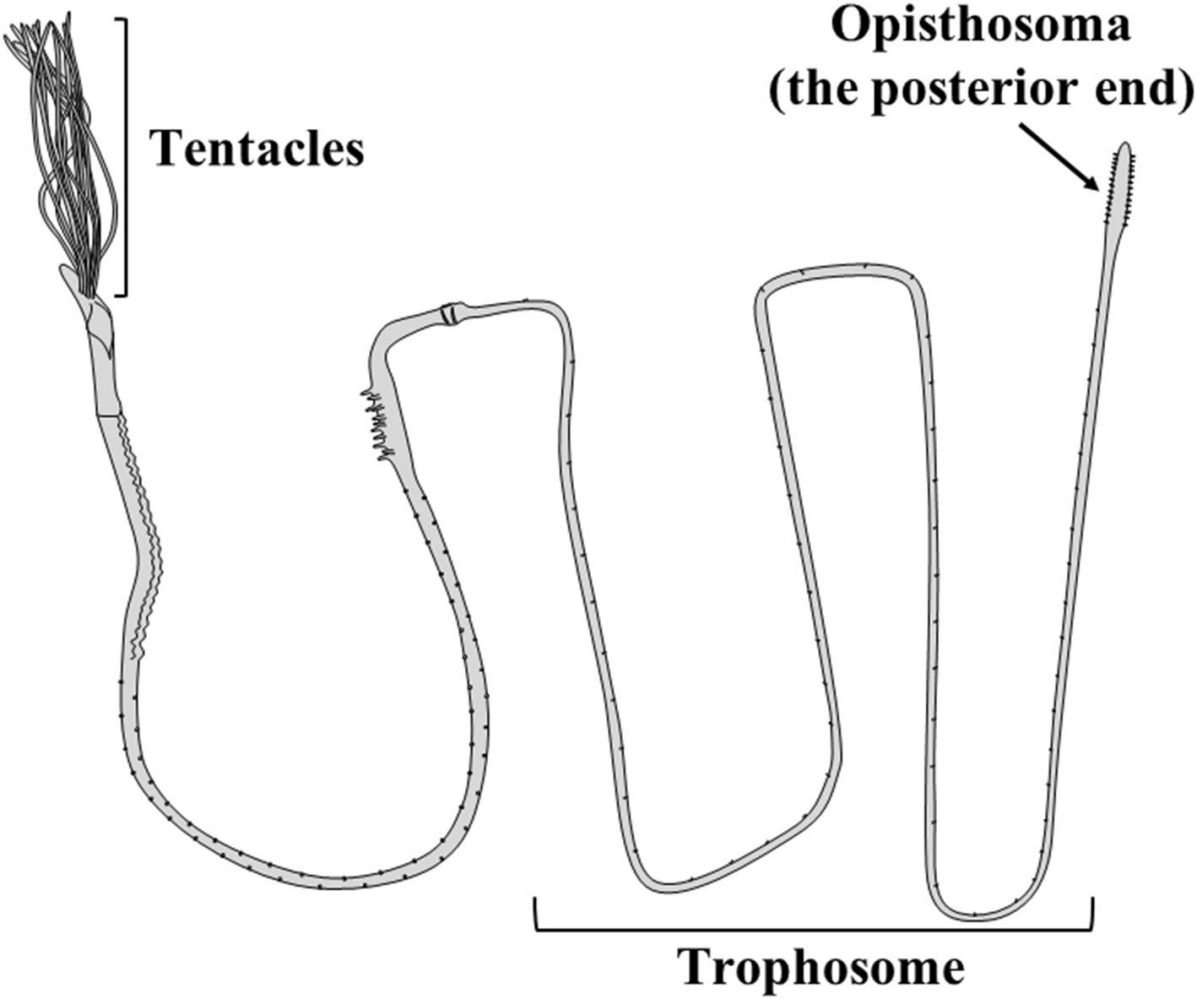
Schematic of the full-length beard worm (*Oligobrachia mashikoi*).

The beard worm is classified as Annelida, Polychaeta, Sabellida, and Siboglinidae^1,2^. The common name “beard worm” originates from the crown of beard-like tentacles at the anterior end of many of these worm species^3^. In addition, these animals share common special features. The beard worm does not have a digestive system consisting of a mouth, gut, and anus, but rather has a special lifestyle utilizing nutrients produced by chemosynthetic bacteria that coexist in the body part called the trophosome^4–6^ (Fig. 1). Most types of beard worms, including *Oligobrachia mashikoi*, inhabit the deep-sea floor (greater than 3000 m)^7^, although there are records of the beard worm, *Siboglinum fiordicum*, being found at shallow depths of 30–35 m in Fanafjorden, Norway^8^. Therefore, it is difficult to observe these worms in their natural habitat in detail because most beard worms live on the deep-sea floor^4^; consequently, many ecological features of beard worms are unresolved.

**Figure 2:**
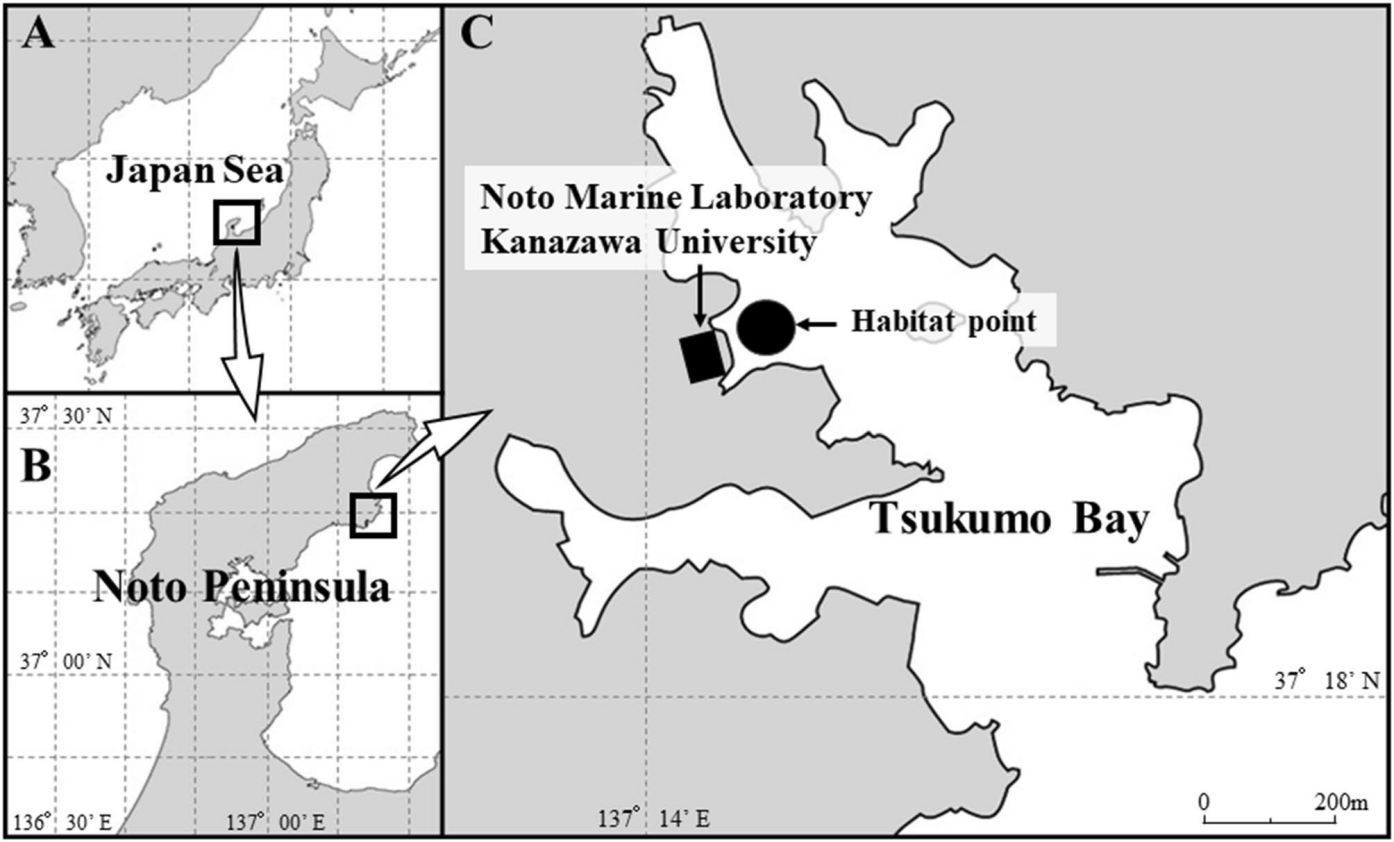
Location of the Sea of Japan (**A**), Noto Peninsula (**B**), and Tsukumo Bay (**C**). Noto Marine laboratory faces the Tsukumo Bay, and the habitat of the beard worm is located just in front of our laboratory. The maps were pictured by both Microsoft® PowerPoint® Microsoft 365 MSO (ver. 2302) and Adobe Photoshop (ver. 19.1.5).

It has been reported that one species of beard worm, *O. mashikoi*, was recorded in 1973 in the shallow muddy seabed (approximately 24.5 m in depth) of Tsukumo Bay of the Noto Peninsula in the Sea of Japan^9^. Thereafter, the whole body was collected, including the posterior end and the “opisthosoma” (Fig. 1)^3^. Furthermore, in front of the Noto Marine Laboratory, a new habitat area of this species was found, which included a wide sea area (3207 m^2^) (Fig. 2)^10^. The shallowest point was 7.3 m and the deepest point was 17.8 m in depth^10^. Therefore, through the ecological survey of *O. mashikoi* living in the shallow sea area of Tsukumo Bay on the Noto Peninsula, it is possible to resolve the ecological aspects of this worm.

**Figure 3:**
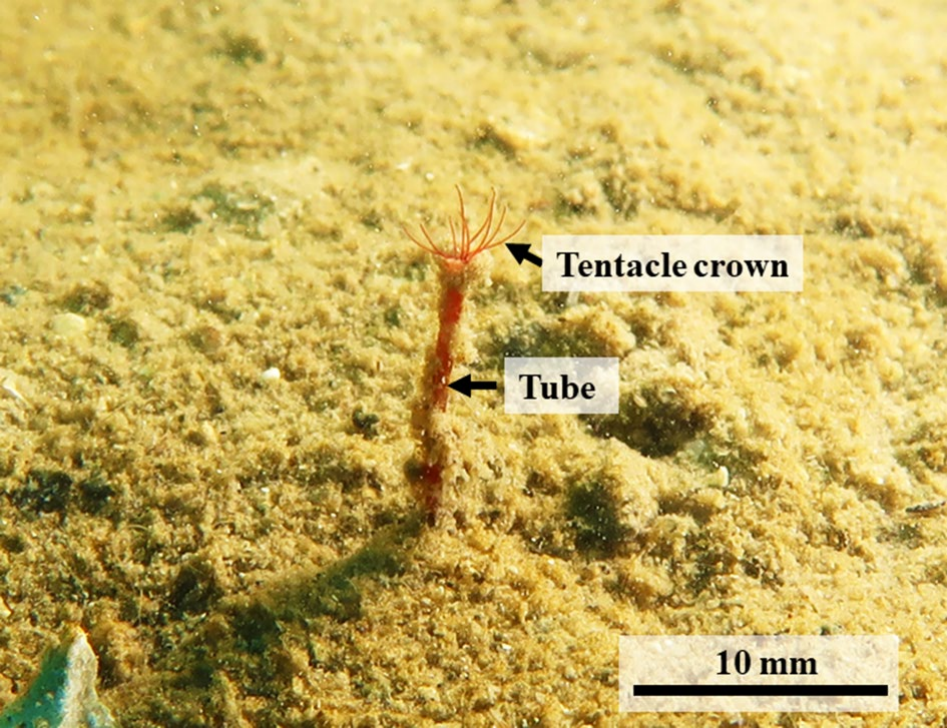
An ecological photograph of the tentacles of a beard worm (*Oligobrachia mashikoi*) expanding out of its inhabited tube.

In the Tsukumo Bay, the sediment was determined to have total sulfide levels of 0.24–0.39 mg/g dry mud^11^. However, the dissolved oxygen concentrations at the bottom level remain high (9.1 ± 0.23 mg/L) throughout the year. Thus, many fish species inhabit the area and appear to be at risk of eating the red tentacles of *O. mashikoi* (Fig. 3). The majority of the Siboglinids below the photic zone are not affected by sunlight. Since *O. mashikoi* appeared to be one of the shallowest dwelling species among Siboglinids and inhabiting depths influenced by diurnal light changes, we hypothesized that the behavioral features, including expanding and contracting the tentacles in response to light, should be present in this species. In addition, the seawater temperature in this shallow area ranged from 10 to 28 °C (Fig. S1) and the tentacle-expanding behavior may be related to water temperature.

In the present study, we performed a scuba diving survey with five quadrats (Fig. S2) to examine tentacle-expanding behavior (Fig. 3) over the course of 7 years to analyze the relationship between seawater temperature or illuminance and tentacle-expanding behavior. Additionally, we utilized a time-lapse camera to examine the dependency of tentacle-expanding behavior of *O. mashikoi* on the time of day. Furthermore, we determined the partial sequence of the photoreceptor, *neuropsin*, and examined the circadian rhythm of *neuropsin* mRNA expression because Neuropsin functions as a photoreceptor in both vertebrates and invertebrates^12–14^. In the Siboglinidae, these results present the first confirmation that the tentacle-expanding behavior of *O. mashikoi* is responsive to light.

## Materials and methods

### Animals

*O. mashikoi* that lives in the Tsukumo Bay of the Noto Peninsula (Fig. 2) was used for ecological observation. For the mRNA expression analysis, 43 individuals were used in the present study.

### Survey of the beard worm *O. mashikoi* in quadrats over a 7-year period

*O. mashikoi* inhabits the seabed at a depth of less than 18 m^10^ which was possible to investigate by scuba diving. In the present study, we surveyed the expanding tentacles of *O. mashikoi* in the Tsukumo Bay (Fig. 2C) by scuba diving over the period of April 2015 to April 2022.

**Figure 4:**
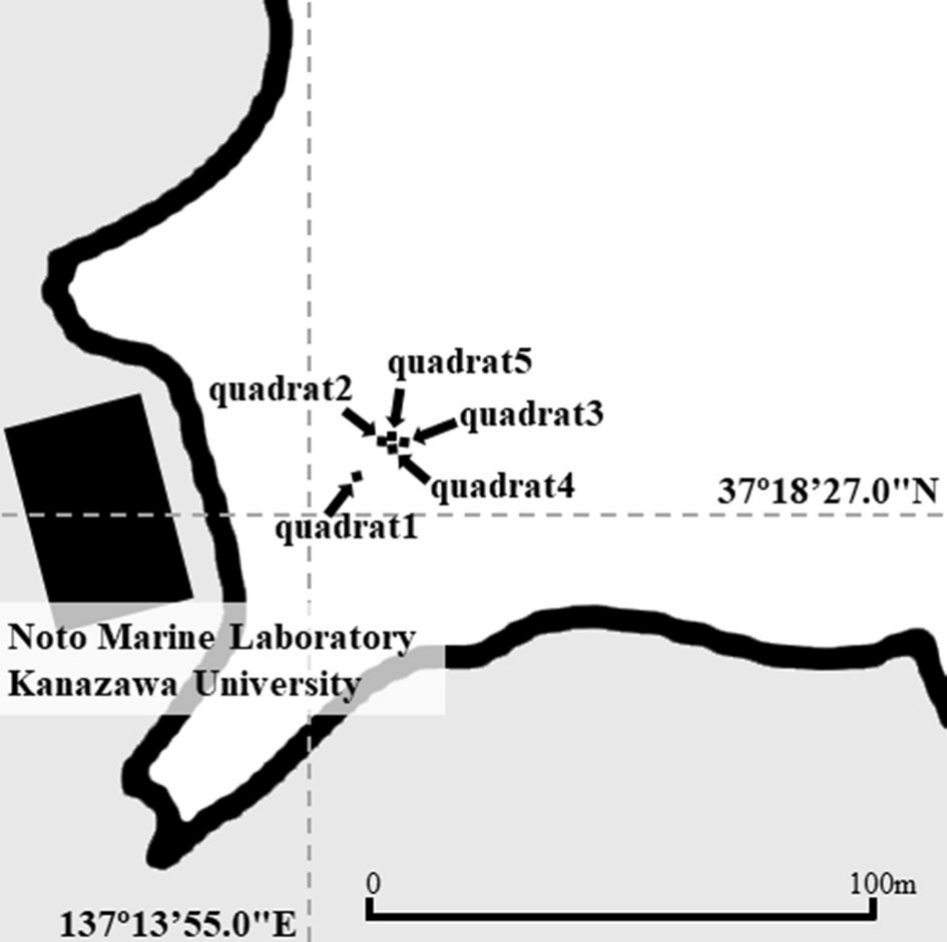
Survey points where the square frames were placed. The water depth at the observation site was approximately 11–13 m, although it changed depending on the tide levels.

To investigate the physiological factors of the tentacle-expending behavior, we used five quadrats (each 1 m^2^) (Fig. S2) at 11–13 m in depth, as shown in Fig. 4, and examined the relationship between tentacle-expanding behavior and seawater temperature or illuminance. These quadrants were randomly selected and the coordinates thereof are indicated in Table S1. We counted the individuals with tentacles expanding from the inhabited tube in each respective quadrat by scuba diving during the day (Supplementary Movie S1). Surveys were conducted from 9:00 to 11:00 am for quadrats 1, 2, and 3 and from 13:00 to 15:00 pm for quadrats 4 and 5 a 7-year period. As there were differences in the number of individuals in each quadrat, the value for each month in the respective quadrat was divided by the average number of individuals for the whole period to calculate the proportion of individuals with expanding tentacles in each month. Seawater temperature and illuminance loggers (HOBO, UA-002-64; Onset Computer Co., MA, USA) (Fig. S3) were placed 30 cm above the seabed at approximately the center of each of the five quadrats during every survey period (August 2015 to April 2022), and measurements were recorded every minute. After every survey, the loggers were removed and the data retrieved. The mean values of the seawater temperature and illuminance during the survey period were recorded as the seawater temperature and illuminance at the time of each survey.

### Evaluation of time-of-day-dependency of the number of *O. mashikoi* with expanding tentacles by a time-lapse camera

To examine the change in the number of *O. mashikoi* with expanding tentacles over the course of a day, time-lapse cameras (Olympus Co., Tokyo, Japan) were placed on the seabed at two points around quadrats (Fig. S4A,B). We counted the number of *O. mashikoi* with expanding tentacles using a time-lapse camera at an hourly rate during two periods (10–15 October 2018 and 29 September–15 October 2020). In our experiment, four or five individuals with tentacles expanding from their tube were continuously observed with a time-lapse camera for 5 days. Because the time-lapse camera utilizes a flash, pictures can be obtained at night. During the scuba diving surveys, underwater photographs were taken to determine whether the tentacles were expanded. To evaluate the tentacle-expanding behavior of each individual, the value was recorded as 1 when the tentacles were expanded and as 0 when the tentacles were not expanded. The values are expressed as the mean ± standard error of the mean (S.E.M.) of four or five independent experiments.

To examine the number of *O. mashikoi* with expanding tentacles under constant dark conditions, we covered the beard worms with a case (Fig. S4C). These experiments were conducted from 24th September to 6th October 2021.

### Determination of the nucleotide sequence of the *neuropsin* photoreceptor from *O. mashikoi* specimens by RNA sequencing

Total RNA from beard worms was prepared using an RNA isolation kit (ISOGEN, Nippon Gene, Toyama), and genomic DNA was removed using an RNase-Free DNase Set (Qiagen). A complementary DNA library was constructed and sequenced with a 150 bp paired-end module using an Illumina NovaSeq 6000 (Illumina, San Diego, CA, USA). The raw sequence reads were deposited in the DNA Data Bank of Japan (DDBJ) under the DDBJ Sequence Read Archive (DRA) accession no. DRA015010. Adaptors and low-quality reads were removed. Subsequently, unigenes were obtained using the Trinity assembling program version r2012-10-05 and v2.13.2. Blast searches were performed on Trinity assemblies, we obtained the nucleotide sequences for elongation factor 1-α (*ef1α*) (accession no. LC730209), glyceraldehyde 3-phosphate dehydrogenase (*gapdh*) (accession no. LC730208), and hypoxanthine–guanine phosphoribosyltransferase 1 (*hprt1*) (accession no. LC730210). Using the RNA-seq data, we searched for a sequence that was highly similar to the nucleotide sequence of *neuropsin* cDNA (accession no. LC726105) in the annelid *Capitella teleta* (accession no. MG710417).

### Analysis of *neuropsin* mRNA expression in the beard worm

*O. mashikoi* specimens were collected by scuba diving (Supplementary Movie S2) to examine the *neuropsin* mRNA expression in this species. In February 2022, *O. mashikoi* was collected from the seabed every 6 h. The worms were placed in clear sample tubes during the day and in light-inhibiting tubes at night. Thereafter, the samples were placed into RNAlater (Sigma-Aldrich, St. Louis, MO, USA) and frozen at − 80 °C until mRNA analysis.

The isolation of total RNA was performed using a commercial extraction kit (RNeasy Plant Mini Kit, Qiagen GmbH, Hidden, Germany). Complementary DNA synthesis was performed using another commercial kit (PrimeScript™ II First-strand cDNA Synthesis Kit, Takara Bio Inc., Otsu, Japan). The gene-specific primers used for *neuropsin* and the primer sequences of *ef1-α*, *gapdh*, and *hprt1* are presented in Table S2. The PCR amplification was performed using a real-time PCR thermocycler (LightCycler^®^ 96, Roche Molecular Systems, Inc., Pleasanton, CA, USA). The *neuropsin*, *ef-1α*, *gapdh*, and *hprt1* were annealed at 60 °C. Relative quantification was performed in accordance with the manufacturer’s protocol using the standard curve method^15,16^. The stock cDNA solution was accurately diluted, and standard curves were prepared using the diluted cDNA. Thus, the relative quantities determined could be compared across multiple plates. The mRNA expression levels were normalized to the expression of *ef1-α* mRNA. *ef1-α* was selected as an internal control gene because its expression level was high and its fluctuation was small compared with the other housekeeping genes (*gapdh* and *hprt1*) in *O. mashik*o*i* (Table S3).

### Statistical analysis

The statistical significance of the correlations between the proportion of individuals with expanding tentacles from their inhabited tubes and seawater temperature or illuminance was examined using simple linear regression analysis. The statistical significance of the difference between the two groups was assessed by the paired *t*-test or Student’s *t*-test. In all data, the selected level of significance was p < 0.05.

## Results

### Quadrat survey of long-term fluctuations in *O. mashikoi* behavior

**Figure 5:**
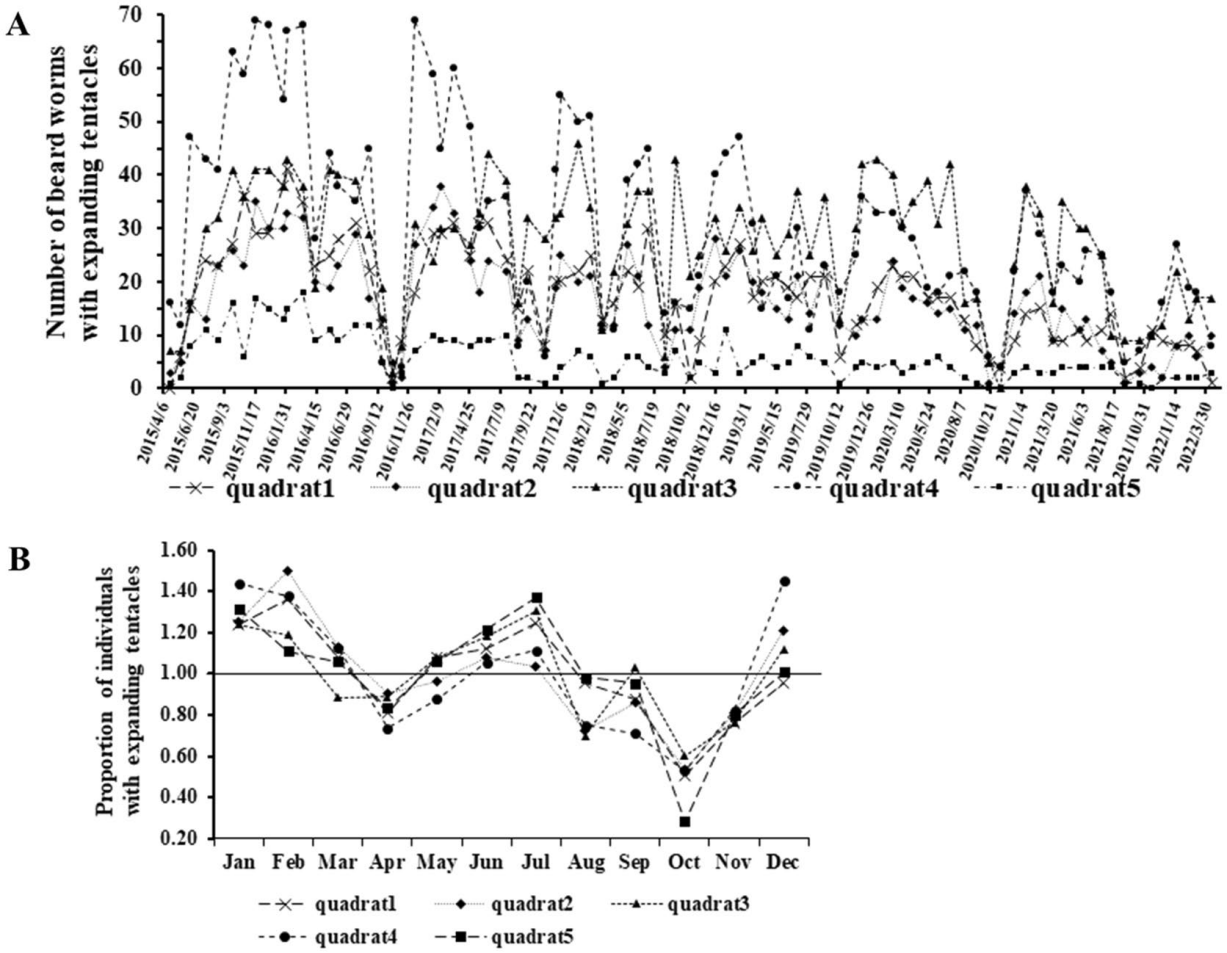
Analysis of beard worms with expanding tentacles from their inhabited tubes by quadrat survey for 7 years. (**A**) Number of beard worms with typical expanding tentacles from their inhabited tubes in each of the five quadrats; (**B**) proportion of individuals with expanding tentacles in each month, calculated by dividing the value for each month by the average number of individuals during the whole period.

A survey extending over a 7-year period was performed in the present study. In this survey, the tentacle-expanding behavior of *O. mashikoi* was recorded on video. A representative video of *O. mashikoi* with expanding tentacles from the inhabited tube is shown in Supplementary Movie S3. The number of *O. mashikoi* with expanding tentacles varied greatly (Fig. 5A). Similar tentacle-expanding behavior occurred in the five different quadrats. The vertical axis shows the proportion of individuals with tentacles expanding out for each month, when the average value for the entire period is set to 1 (Fig. 5B). Many individuals were found to have tentacles expanding from their inhabited tubes in January, February, and July. In contrast, in April, the number of individuals with tentacles expanding out decreased and in particular, the *O. mashikoi* individuals with expanding tentacles were reduced in October.

**Figure 6:**
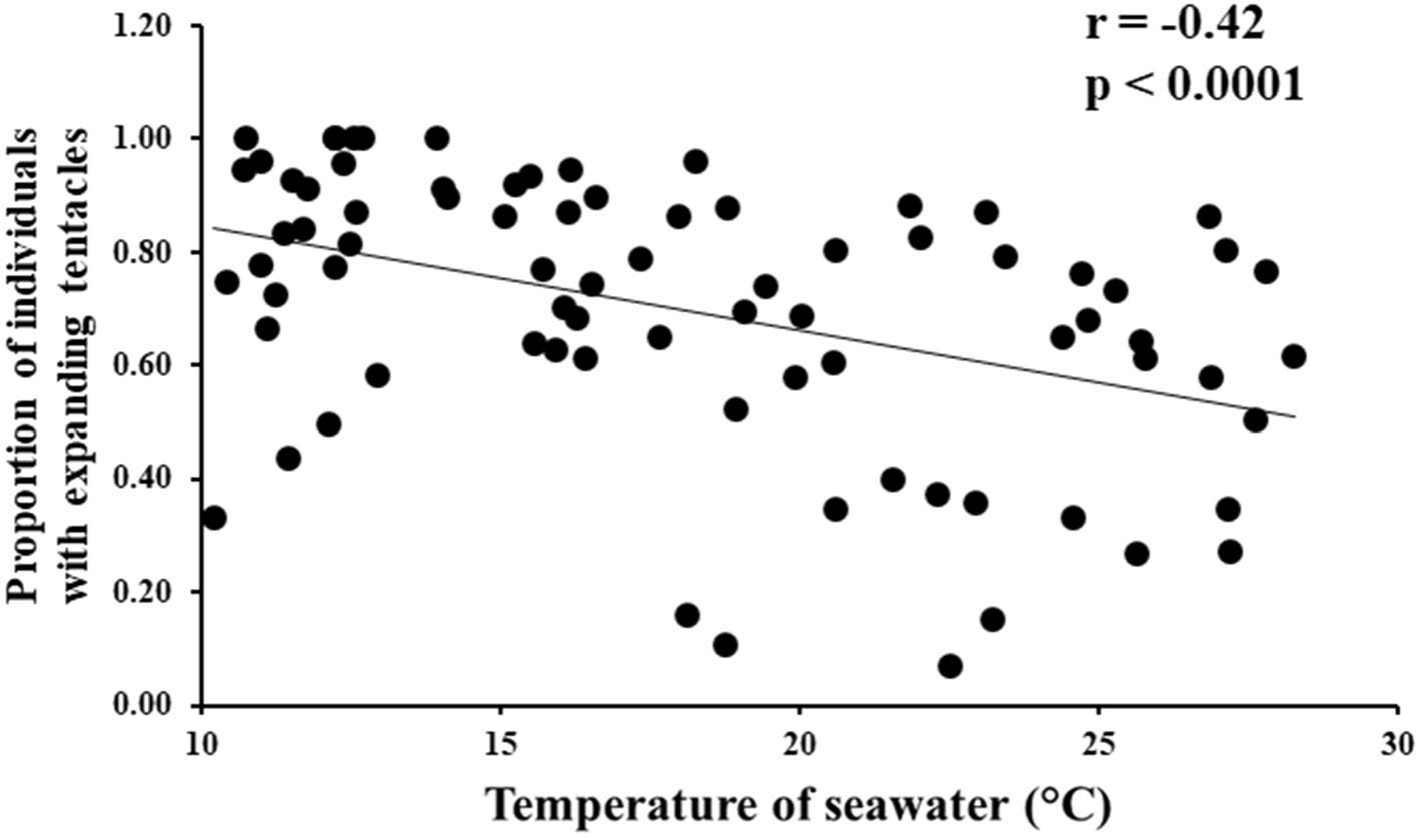
The relationship between the proportion of individuals with expanding tentacles from their inhabited tubes and seawater temperature.

**Figure 7:**
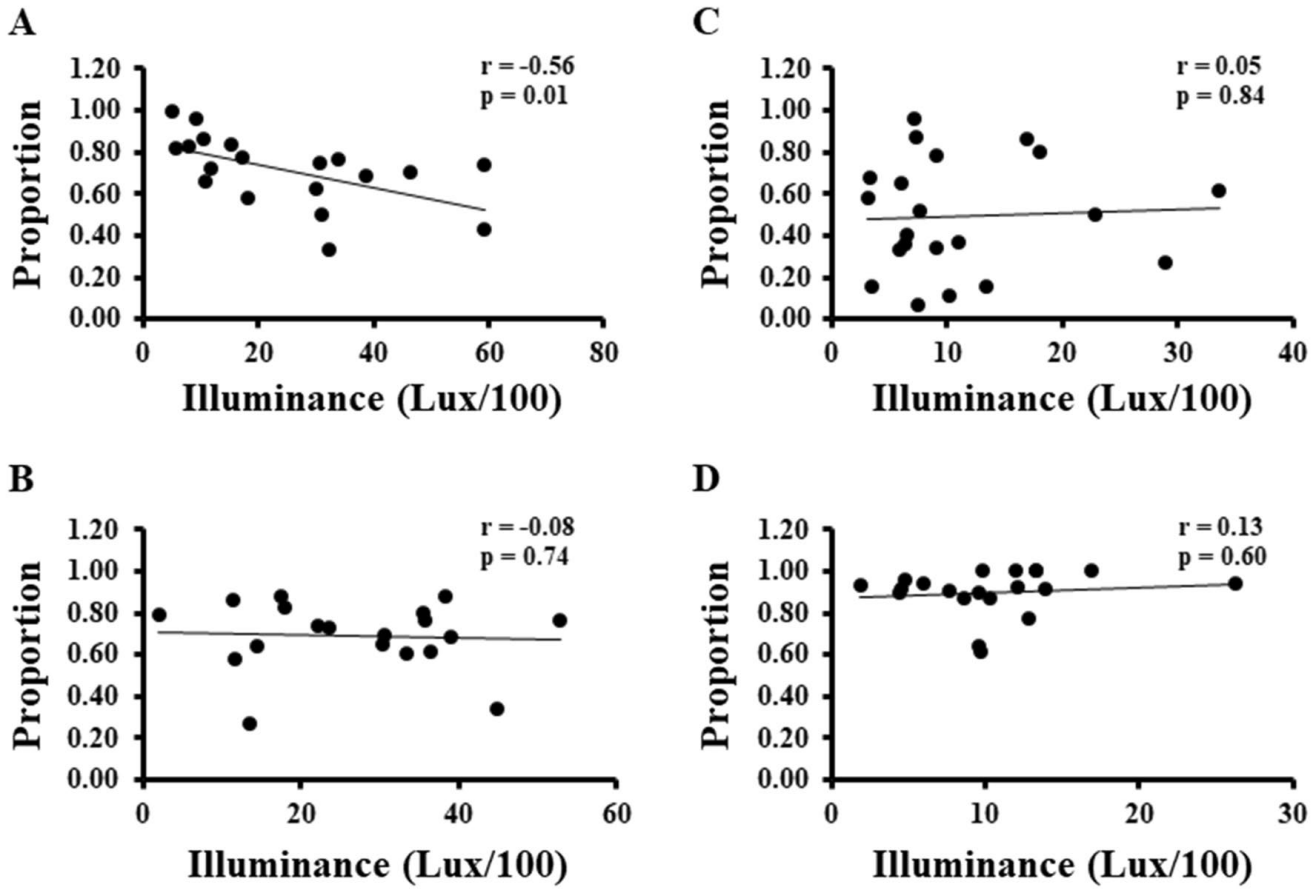
The relationship between the proportion of individuals with expanding tentacles from their tubes and illuminance in spring (**A**), summer (**B**), autumn (**C**), and winter (**D**). (**A**) March to May; (**B**) June to August; (**C**) September to November; (**D**) December to February.

In addition, we found that the seawater temperature and proportion of individuals with expanding tentacles were negatively correlated throughout the year (Fig. 6). Furthermore, the relationship between the illuminance and proportion of individuals with expanding tentacles was significantly negative correlated in spring (March to May) (Fig. 7A). No significant correlations were found for the other seasons (Fig. 7B–D).

### Time-lapse camera survey of diurnal fluctuations in *O. mashikoi* behavior

**Figure 8:**
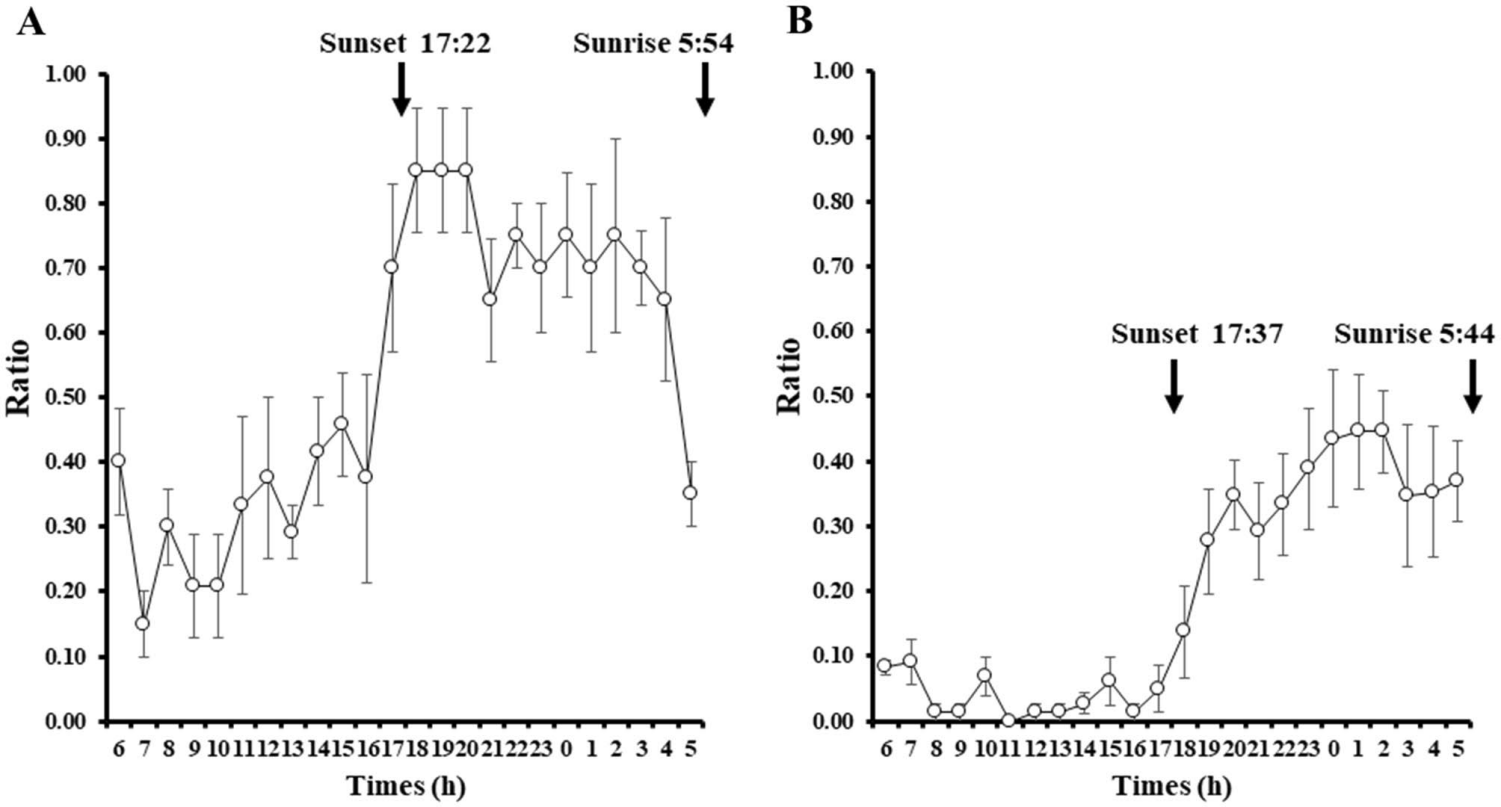
Evaluation of time-of-day-dependent changes in the number of *Oligobrachia mashikoi* thrusting tentacles from their tubes by a time-lapse camera. The time-lapse camera was placed in the vicinity of the square frame in Fig. 4, where several *O. mashikoi* could be observed. (**A**) *O. mashikoi* was analyzed at each time point indicated to evaluate whether they thrusted tentacles from their tubes on October 10–15 2018. The Y-axis presents the ratio of the number of times that tentacle-thrusting behavior was observed to the number of analyses (n = 4–6). The values are the mean ± S.E.M. of four independent experiments. (**B**) *O. mashikoi* was analyzed at each time point indicated to evaluate whether they thrusted tentacles from their tubes between 29 September and 15 October 2020. The Y-axis presents the ratio of the number of times that tentacle-expanding behavior was observed to that of analyses (n = 9–15) for each individual. The time of sunset and sunrise are indicated by their respective arrows. The values are the mean ± standard error of the mean (S.E.M.) of five independent experiments.

The diurnal fluctuation of *O. mashikoi* was examined in the present study. For each individual photographed, the average value was calculated for each hour during the period of photography for which the tentacles expanded from their inhabited tubes. The black line indicates the average proportion of the tentacles expanding for all individuals. After sunset, the proportion of tentacles expanding out of the inhabited tube increased, whereas the proportion of tentacles expanding out decreased after sunrise (Fig. 8A,B).

**Figure 9:**
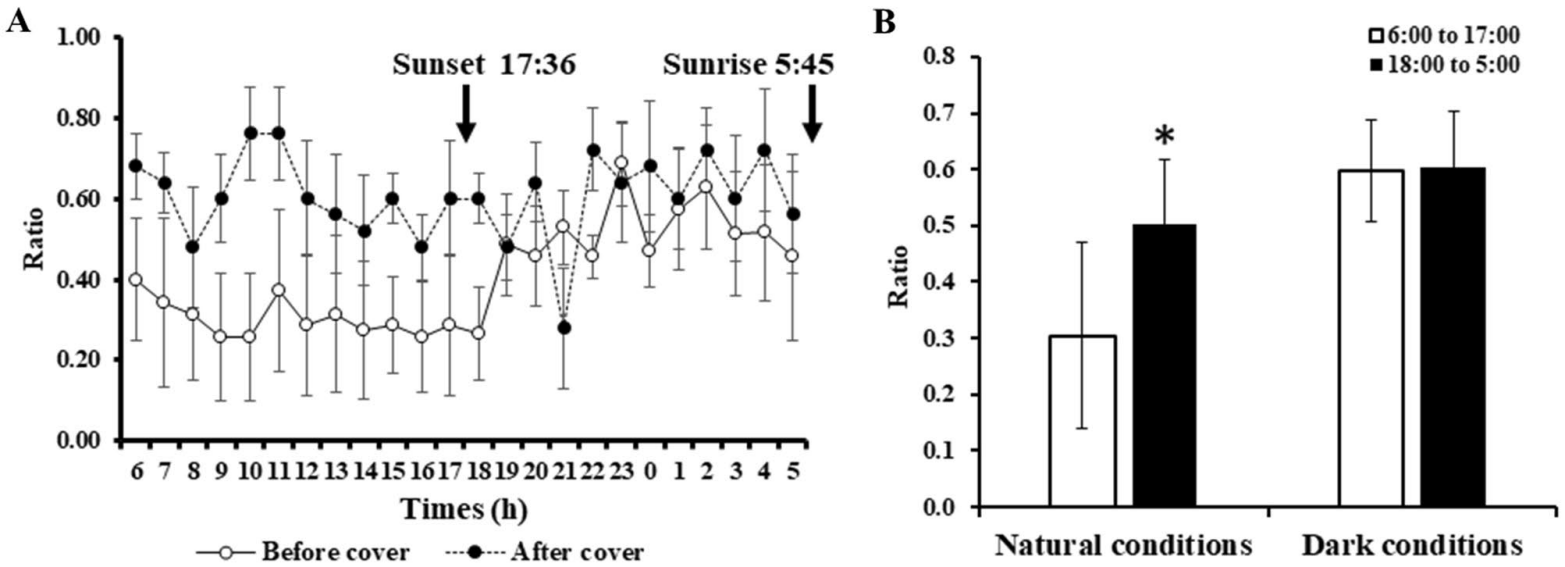
Influence of light prevention on the tentacle-thrusting behavior of *Oligobrachia mashikoi* in the inhabited seabed area. (**A**) *Oligobrachia mashikoi* was analyzed at each time point indicated to evaluate whether they thrusted tentacles from their tubes. The Y-axis presents the ratio of the number of times that tentacle-thrusting behavior was observed to the number of analyses (n = 5 to 8). The values are the mean ± S.E.M. of five independent experiments. Experiments were performed on September 24–October 6, 2021. Animals were covered with a case (Fig. S3C) to prevent them from being exposed to light during daytime on October 1–6, 2021 (black circle), while they were not covered on September 24–October 1, 2021 (white circle). The time of sunset and sunrise are indicated by their respective arrows. (**B**) The average values of the ratios of the number of times that tentacle-thrusting behavior was observed to the number of analyses at each of time point between 6:00 and 17:00 (white bar) or 18:00 to 5:00 (black bar) in (**A**) are described. The values are the mean ± standard error of the mean (S.E.M.) of five independent experiments. Animals were covered with a case from 1st to 6th October, 2021 (dark conditions), and not covered from 24th September to 1st October, 2021 (natural conditions). A paired *t*-test was performed to examine the statistical significance of the changes between daytime and nighttime in the same individuals. *p < 0.05.

The average rate of tentacle-expanding behavior from the five beard worms before and after the cover was placed over their inhibited seabed area is shown in Fig. 9. For the uncovered specimens under natural conditions, an increase in the tentacle-expanding behavior of individuals was observed during nighttime (6:00 pm to 5:00 am) compared to daytime (6:00 am to 5:00 pm) (Fig. 9). Under constant dark conditions with the cover placed over the individuals, a difference between the night and day regarding the tentacle-expanding behavior was absent (Fig. 9).

### Expression of neuropsin mRNA in the beard worm

**Figure 10:**
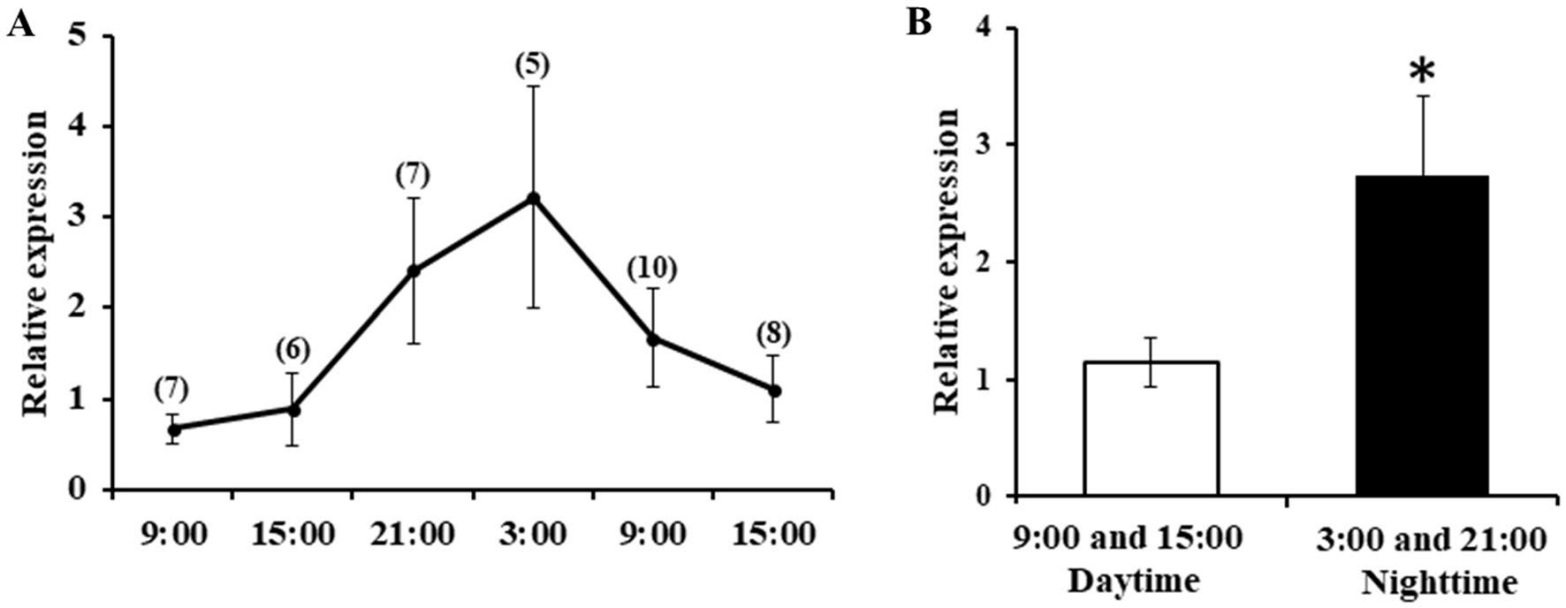
mRNA expression profiles of *neuropsin* in *Oligobrachia mashikoi*. (**A**) Beard worms were collected from the seabed every 6 h; (**B**) expression levels of *neuropsin* mRNA during daytime (9:00 and 15:00) and nighttime (21:00 and 3:00). The statistical significance between the daytime and nighttime groups was assessed using an independent sample *t*-test. The number of individuals at each hour is shown in parentheses. *p < 0.05.

*O. mashikoi* was collected from the seabed every 6 h by scuba diving (Supplementary Movie S2). Expression levels of *neuropsin* mRNA were determined for each time point. The expression of *neuropsin* mRNA was the highest at 3:00 am (Fig. 10A). Moreover, *neuropsin* mRNA expression in the night sampled beard worms (9:00 pm to 3:00 am) was significantly higher than in the day sampled beard worms (9:00 am to 3:00 pm) (Fig. 10B).

## Discussion

The beard worm *O. mashikoi*, was found to inhabit the shallow muddy seabed in Tsukumo Bay of the Noto Peninsula^3,9^. The shallow-water habitat of *O. mashikoi* allowed us to examine the effect of environmental stimuli on *O. mashikoi* in this study, including the effect of light–dark cycles.

For the first time, we succeeded in capturing a video of the beard worm expanding its tentacles from of its inhabited tubes under natural conditions (Supplementary Movie S3). This is uniquely achievable for species that can be surveyed by scuba diving. Furthermore, over the course of a 7-year quadrat survey, we found that the beard worm possessed a seasonal pattern of tentacle-expanding behavior (Fig. 5). The 7-year survey period revealed that during some months of the year, two reductions in the abundance of tentacle-expanded individuals were reported—in April and October. The spring (April) drop may be related to increased light sensitivity. On the other hand, the decrease in the number of tentacle-expanding individuals from the inhabited tubes in October may be related to the spawning season of this animal (Fig. S5). Spawning may cause some physiological changes that limit the behavior of tentacle protrusion from the inhabited tubes. We also found that sea water temperature and illuminance are environmental factors that influence the tentacle-expanding behavior of *O. mashikoi*. From the observed correlation between seawater temperature and tentacle-expanding behavior from the inhabited tube (Figs. 6, 7), it is possible that sensitivity to light may have increased in spring because the seawater temperature is the lowest in the area where the *O. mashikoi* lives throughout the year (Fig. S1). Other factors may be involved, and we would like to conduct a detailed analysis of the inorganic and organic components of seawater in future studies.

We found that the number of *O. mashikoi* with expanding tentacles increased during the night (Fig. 8). In addition, by preventing *O. mashikoi* from exposure to light the differences in the number of individuals with expanding tentacles was eliminated (Fig. 9). Collectively, these findings indicate that *O. mashikoi* detects and uses light signals to control its tentacle-expanding behavior. Beard worms expanding their tentacles from their inhabited tubes to breath, which may increase their risk of being captured by predators. It is tempting to speculate that *O. mashikoi* uses light signals to escape from its predators during day when they are active. We intend to continue time-lapse camera surveys to elucidate the relationship between tentacle-expanding behavior and predators.

An important issue raised by the current study concerns the identity of the phototransducing molecules responsible for the light-dependent inhibition of the tentacle-expanding behavior of *O. mashikoi*. This study identified genes with sequence similarity to invertebrate genes coding for *neuropsin* in *O. mashikoi*. The *neuropsin* has been reported to function as a photoreceptor in the chick (*Gallus gallus*) and Atlantic horseshoe crab (*Limulus polyphemus*). In the chick, *neuropsin* was indicated to be the photoreceptor regulating photoperiodism, which is involved in seasonal reproduction control^13^. In the Atlantic horseshoe crab, *neuropsin* was proposed to function as the lateral and ventral eye photoreceptors through the collaboration with other opsin genes^12^. Based these findings, we speculate that *neuropsin* in *O. mashikoi* functions as a photoreceptor involved in the light-dependent control of its tentacle-expanding behavior by passing light through the inlet hole or tube wall, although it should be stressed that photoreceptors other than *neuropsin* are also involved in the control. This expression of the photoreceptor molecule *neuropsin* further supports the possibility that *O. mashikoi* can respond to light. Another type of photoreceptor has been reported in a Pogonophore^17,18^. We will examine the involvement of this photoreceptor in the light-dependent control for tentacle-expanding behavior in *O. mashikoi* in future studies.

Records show that the beard worm, *Siboglinum fiordicum,* has been found at the shallow depth of 30–35 m in Fanafjorden, Norway^8^. Beard worms collected in Norway possess a single tentacle, and unlike *O. mashikoi*, do not have multiple tentacles. As *O. mashikoi* possesses multiple tentacles, it is possible to observe its tentacles expanding from the inhabited tubes through scuba diving surveys. Thus, we strongly believe that *O. mashikoi* is a model species for which diving surveys could be conducted and its ecology can be observed. It is believed that the tentacles expand from the inhabited tubes to take in oxygen for respiration and to supply hydrogen sulfide for chemosynthesis by the symbiotic bacteria because the hemoglobin in *O. mashikoi* possesses binding activity for oxygen and sulfide^19,20^. However, no studies have demonstrated that the tentacles of *O. mashikoi* absorb oxygen or hydrogen sulfide from the environmental seabed. Since this species lives in shallow-water, it is possible to conduct a variety of studies including respiratory function to elucidate the function of their tentacles.

*O. mashikoi* was found in the sea floor at depths of 100 and 200 m in the Sea of Japan^21,22^, suggesting that *O. mashikoi* has a shallow-water habitat. Notably, *O. mashiko*i with eggs was found in October (Fig. S5), which is their breeding season, during the 7-year survey period. This finding, together with the fact that its lifespan appears to be short, indicates that *O. mashikoi* has adapted to the shallower sea floor environment of Tsukumo Bay. Considering this, our results suggest that their intrinsic ability to detect and utilize light may have facilitated the adaptation of *O. mashikoi* to the shallow sea floor environment by conferring a survival advantage.

## Conclusion

One species of beard worm, *O. mashikoi*, which specifically inhabits the shallow sea area of the Tsukumo Bay on the Noto Peninsula in the Sea of Japan, is a unique species that can be studied ecologically and physiologically by scuba diving. Our results are the first to show that in Siboglinidae, *O. mashikoi* follows a pattern of light response regarding their tentacle-expanding behavior. Although most types of beard worms, including *O. mashikoi*, inhabit the deep-sea floor (greater than 3000 m)^7^, the range of depths they occupy is vast^8^. Thus, we speculate that the light response of the beard worm *O. mashikoi* contributes to the expansion of the depths at which this species can inhabit.

## Supplementary Information

Supplementary Information.

Supplementary Video 1.

Supplementary Video 2.

Supplementary Video 3.

## Data Availability

The RNA-sequencing data have been deposited in the DNA Data Bank of Japan (DDBJ; https://www.ddbj.nig.ac.jp/index-e.html) with the DRA accession number of DRA015010. The *neuropsin*, *gapdh*, *ef1α*, and *hprt1* sequences’ data have been deposited in the DDBJ with the LC accession numbers of LC726105, LC730209, LC730208, and LC730210, respectively.
