# Supplementary material for: Adaptation to the shallow sea floor environment of a species of marine worms, Oligobrachia mashikoi, generally inhabiting deep-sea water
Source: Sci Rep. 2023 Apr 18;13:6299. doi: 10.1038/s41598-023-33309-6 (PMC10113264; doi:10.1038/s41598-023-33309-6)
Supplement: Supplementary file 1 — Supplementary Information. [file 41598_2023_33309_MOESM1_ESM.pdf]

Supplementary data for

Adaptation to the shallow sea floor environment of a species of marine worms, *Oligobrachia mashikoi*, generally inhabiting deep-sea water

Shouzo Ogiso, Kazuki Watanabe, Yusuke Maruyama, Hiroshi Miyake, Kaito Hatano, Jun Hirayama, Atsuhiko Hattori, Yukina Watabe, Toshio Sekiguchi, Yoichiro Kitani, Yukihiro Furusawa, Yoshiaki Tabuchi, Hajime Matsubara, Mana Nakagiri, Kenji Toyota, Yuichi Sasayama, and Nobuo Suzuki

This pdf file includes:

Supplementary Figure S1 to S5
Supplementary Table S1 to S3

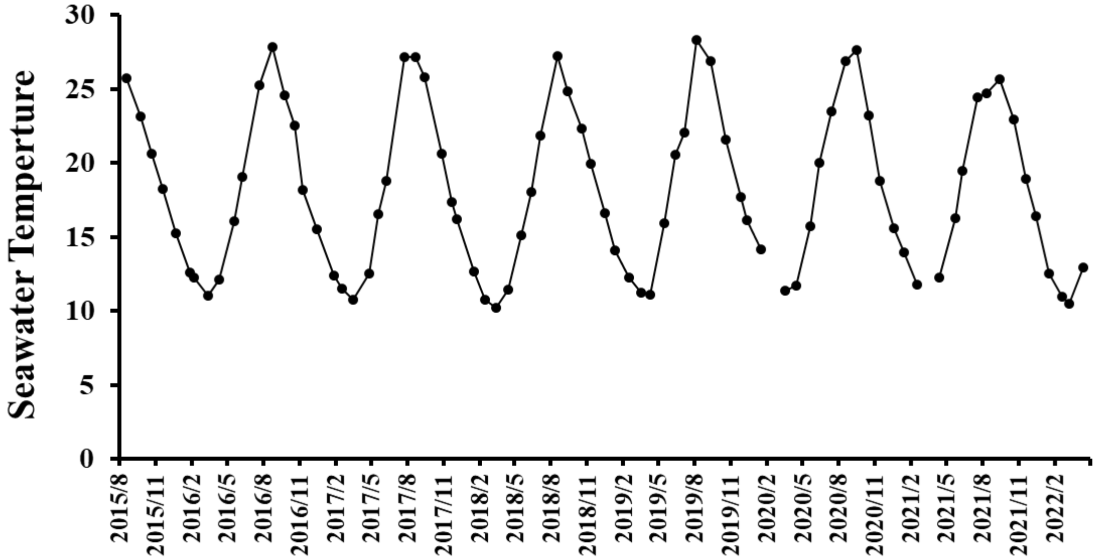

Supplementary Figure S1. Monthly changes in seawater temperature in Tsukumo Bay over 7 years.

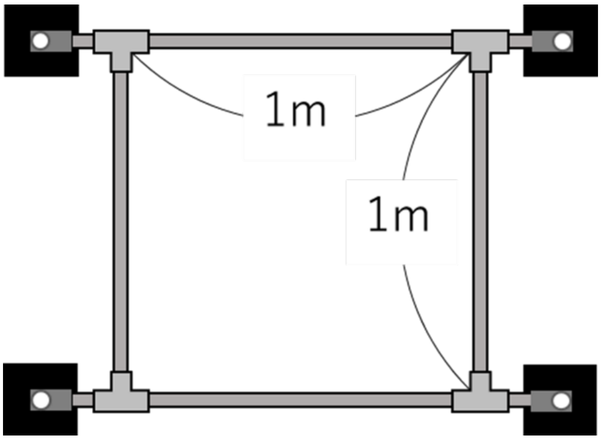

Supplementary Figure S2. The size of the square frame used for the quadrat survey.

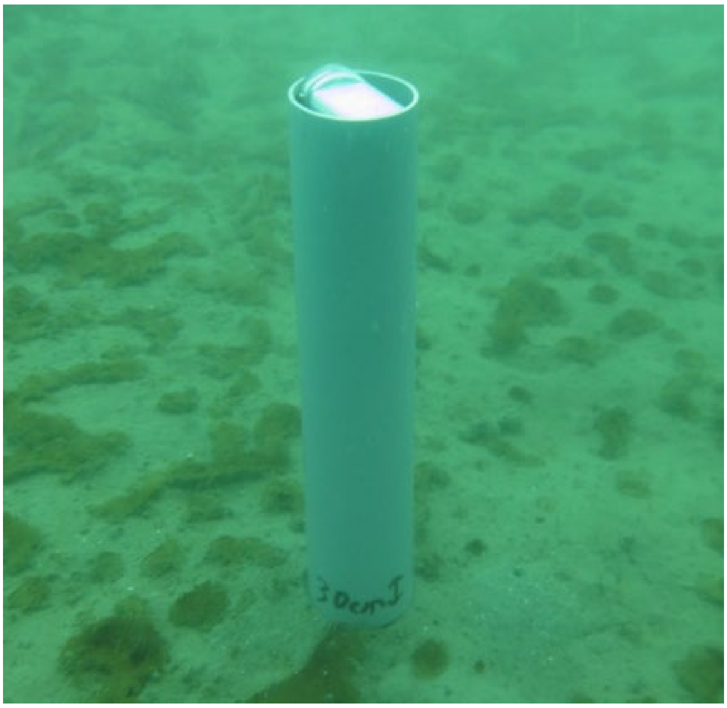

Supplementary Figure S3. Seawater temperature and illuminance loggers used for scuba diving surveys.

Seawater temperature and illuminance loggers (HOBO, UA-002-64; Onset Computer Co., MA, USA) were placed at 30 cm above the seabed at approximately the center of five quadrats during every survey period. Seawater temperature and illuminance were recorded every minute.

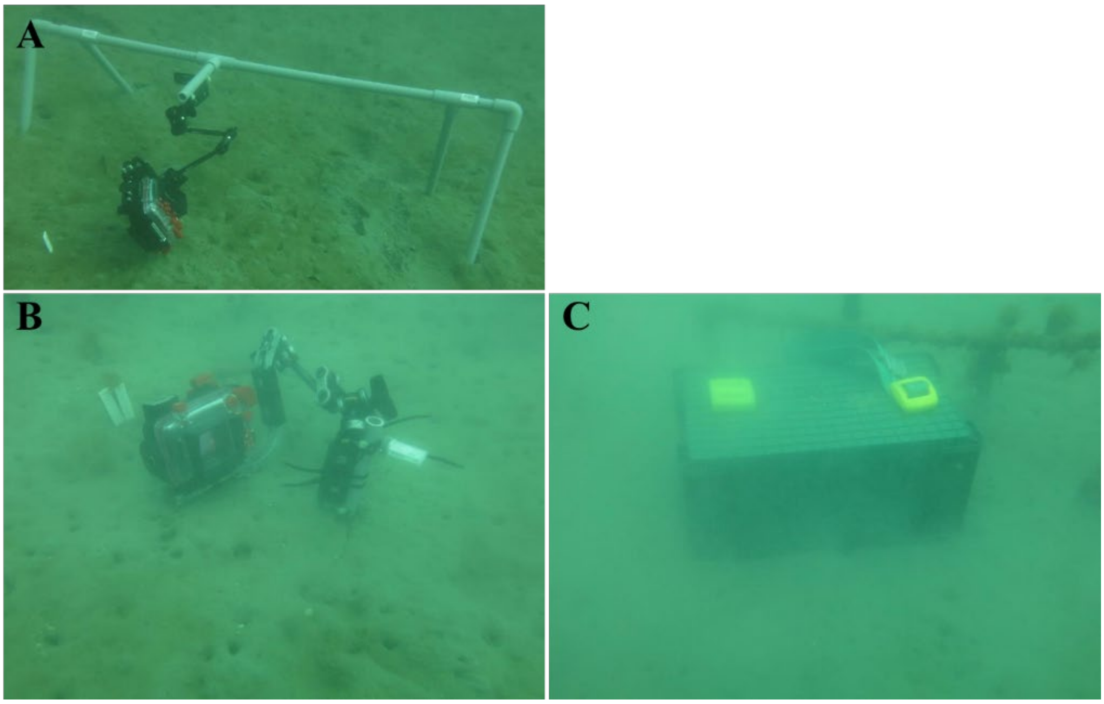

Supplementary Figure S4. Time-lapse cameras used in this study.

Time-lapse cameras (Olympus Co., Tokyo, Japan) were placed on the seabed at two points (A and B) where several *Oligobrachia mashikoi* could be observed. These points were placed around quadrat survey area. The influence of the prevention of light exposure during the daytime on the tentacle-expanding behavior of *O. mashikoi* was examined by covering the time-lapse camera with a case (C).

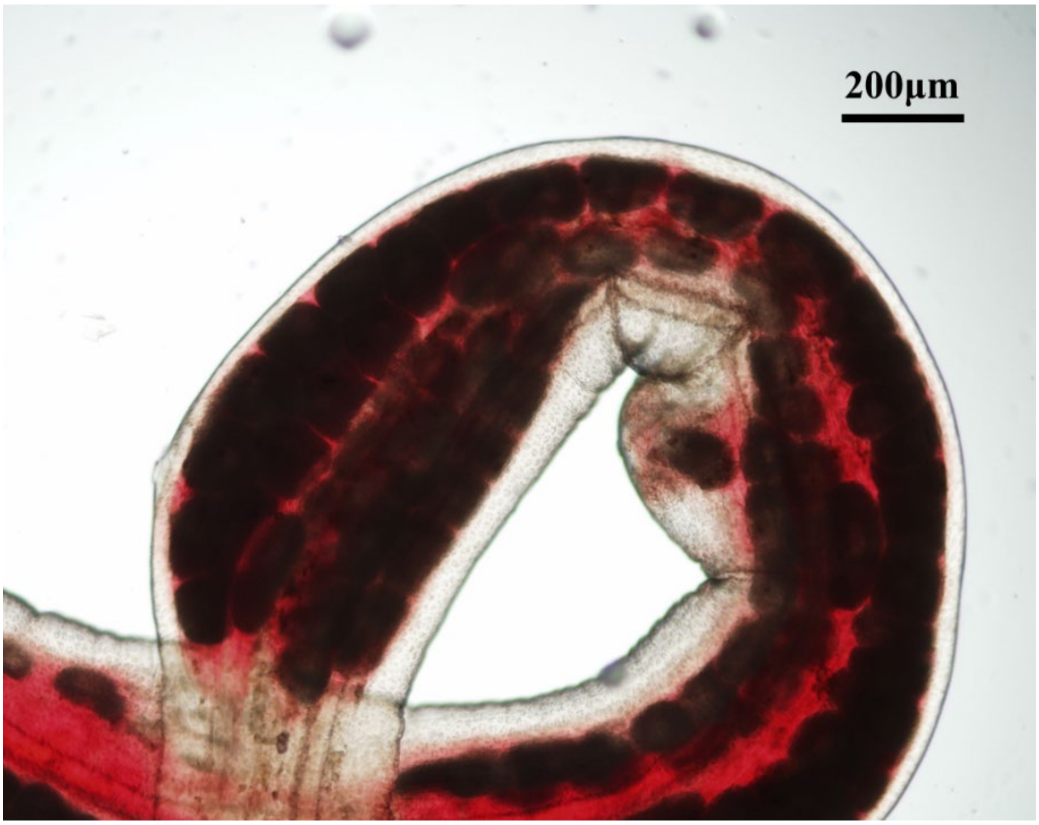

Supplementary Figure S5. Photograph of beard worm with eggs on October 3, 2016.

Supplementary Table S1. The latitude and longitude of respective quadrant used in the present study.

quadrad	latitude	longitude
1	37º18'27.1"N	137º13'55.2"E
2	37º18'27.3"N	137º13'55.4"E
3	37º18'27.3"N	137º13'55.6"E
4	37º18'27.2"N	137º13'55.5"E
5	37º18'27.3"N	137º13'55.5"E

Supplementary Table S2. Primer sequences for *neuropsin, ef1-α, gapdh,* and *hprt1* genes used for quantitative RT-PCR.

Name	Forward primer	Reverse primer	Accession No.
neuropsin	TGTTCGGCAACACGTCGGTGAT	TCACAACGGCAGCAGACGACAA	LC726105
ef1-α	GAACGCCAACAACGCATGCTCA	AAGGCAGAGCGTGAACGTGGTA	LC730209
gapdh	CAATGCCAGCGCCAGCATCAAA	TGGAATGGCTTTCCGCGTTCCT	LC730208
hprt1	CTTCTCCCGTCGTTGATGTGTCCA	TGCATCCCGAGGCATTACAACGAA	LC730210

ef1-α: elongation factor 1-α; gapdh: glyceraldehyde-3-phosphate dehydrogenase; hprt1: hypoxanthine phosphoribosyltransferase 1

Supplementary Table S3. Expression of housekeeping genes (*ef1-a*, *gapdh* and *hprt1*) in *Oligobrachia mashikoi*.

	ef1-α	*gapdh*	*hprt1*
n	43	43	43
mean	4354.5	8044.8	6088.6
S.E.M.	492.1	695.4	1065.6
